# Short arm splints for wrist stabilization: A mechanical material test and cadaveric radiography study

**DOI:** 10.1002/jeo2.70065

**Published:** 2024-10-26

**Authors:** Hans Christian Rasmussen, Maya Bang, Johanne Gade Lilleøre, Josephine Olsen Kipp, Lars Lindgren, Annemarie Brüel, Mats Bue, Mads Kristian Duborg Mikkelsen, Jesper Skovhus Thomsen, Maiken Stilling

**Affiliations:** ^1^ Orthopaedic Research Laboratory Aarhus University Hospital Aarhus Denmark; ^2^ Department of Orthopaedic Surgery Aarhus University Hospital Aarhus Denmark; ^3^ Department of Radiology Aarhus University Hospital Aarhus Denmark; ^4^ Department of Biomedicine Aarhus University Aarhus Denmark

**Keywords:** cadaveric setup, fracture stabilization, short arm splint, three‐point bending test, wrist immobilization

## Abstract

**Purpose:**

Documentation of the wrist stabilizing effect and mechanical properties of common splinting materials is warranted to support evidence‐based condition‐specific recommendations for wrist immobilization. The objectives of this study were to assess the wrist stabilizing properties of volar and dorsal short‐arm splints made of four different materials and evaluate the mechanical properties of the splinting materials.

**Methods:**

Dorsal and volar short arm splints made of plaster of Paris (PoP) (eight layers), Woodcast (2 mm, rigid vented), X‐lite (classic, two layers) or a 3D‐printed material (polypropylene) were sequentially mounted on 10 cadaveric arm specimens and fixed in a radiolucent fixture. This enabled the evaluation of maximum wrist flexion and extension relative under an orthogonal load of 42 N via radiographic images. In addition, a three‐point bending test was performed on ten sheet duplicates of each of the four splinting materials.

**Results:**

When applied as a volar splint, PoP had the highest capability to resist wrist flexion and extension. However, when applied as a dorsal splint, Woodcast exhibited a lower wrist flexion and a similar wrist extension. The 3D‐printed splints—both volar and dorsal—showed the highest mean wrist flexion and extension. The mechanical properties of the Woodcast, X‐lite and 3D‐printed splinting materials were very similar. PoP exhibited distinct properties with a stiffness of 146 (95% confidence interval [CI]: 120–173) N/mm and a deflection at *F*_max_ of 0.6 (95% CI: 0.5–0.7) mm compared to ≤7.7 (95% CI: 7.4–7.9) N/mm and ≥20 (95% CI: 18–22) mm for the other materials.

**Conclusion:**

PoP displayed better wrist stabilizing properties and material stiffness than Woodcast, X‐lite and 3D‐printed polypropylene. When considering wrist stabilizing properties, PoP may still prove to be the preferred choice for wrist immobilization.

**Level of Evidence:**

Not applicable.

**Abbreviations**
3D: three‐dimensional
95% CI: 95% Confidence interval
95% LoA: 95% Limits of agreement
*F*_max_
: force to fracture (N)
ICC: intraclass correlations coefficient
MD: mean difference
Ns: statistical non‐significance (*p* > 0.05)
PoP: plaster of Paris
Stiffness: extrinsic flexural rigidity (N/mm)
Δx at *F*_max_
: deflection at maximum extrinsic fracture load (mm)

## INTRODUCTION

Conventionally, temporary immobilization of the wrist has been achieved by the use of a cast or a splint [8]. A splint may be relevant for several conditions, such as painful tenosynovitis, conservative treatment of fractures, and following surgery, for example, fracture, arthrodesis or wrist arthroplasty [16, 24]. However, the degree of immobilization required for the different conditions is not documented. Likewise, the wrist‐stabilizing effect of different splinting materials is scarcely evaluated and compared in the literature [13].

Historically, plaster of Paris (PoP) has been the material of choice for wrist immobilization, particularly for fracture stabilization of distal radius fractures [14]. Thermoplastic composite materials have become widely adopted due to their ease of use and reshapeable properties, which may alleviate pressure‐related problems [15]. Moreover, technological advancements and emphasis on patient satisfaction have pushed for the use of three‐dimensional (3D)‐printed splints, customized to the individual patient. Biomechanical studies have examined the stabilizing effects of thermoplastic braces, fibreglass and 3D‐printed casts in experimental distal radius fracture models [15, 25, 29]. However, there is a lack of data on the wrist‐stabilizing properties of short‐arm splints as well as the mechanical properties of common splinting materials [13, 16]. Addressing these gaps is warranted, as greater immobilization holds the potential to improve bone healing outcomes.

The aims of the present study were to (1) evaluate the extent of wrist flexion and extension achieved with volar and dorsal short arm splints made from four different splinting materials (X‐lite [classic, two layers], Woodcast [2 mm, rigid vented], PoP [eight layers] and 3D‐printed [polypropylene]) under constant load in a standardized cadaveric setup and (2) determine the mechanical properties of the four splinting materials using a three‐point bending test.

## METHODS

The study was conducted at the Department of Clinical Medicine, Aarhus University Hospital, Denmark, and carried out in agreement with national and international law and with approval by the Central Denmark Region Committees on Health Research Ethics (license No. 1‐10‐72‐144‐23). Mechanical testing of the splinting materials was performed at the Department of Biomedicine, Aarhus University, Denmark.

### Specimens

Ten arm specimens (fresh freezing preparation) obtained from five human donors were included in the study. The specimens were provided by the Department of Biomedicine, Aarhus University. The arm specimens were obtained from two female (aged 65 and 72 years) and three male (aged 83, 85 and 90 years) donors. The arm specimens included the elbow and the distal part of the brachium, ensuring that all flexor and extensor tendons were attached to their respective origins. Before inclusion in the study, the donors were fully pseudonymized, and conventional X‐ray images of the arm specimens were obtained to confirm the absence of bone malformations, prostheses and osteosyntheses. Prior to inclusion and on the first testing day, all arm specimens were thawed slowly at room temperature for 24 h. Then, a senior hand surgeon assessed the range of motion in the wrist and fingers of the arm specimens. Only arm specimens with a normal range of motion were included. Testing was performed on three consecutive days to accommodate splint hardening before testing. All arm specimens were tested sequentially with all splinting materials applied as volar and dorsal short arm splints. To ensure preservation, the arm specimens were stored at 5°C between testing days and allowed to heat to room temperature before testing.

### Splinting materials

Four different splinting materials (Table 1) were used to create both a volar and a dorsal short‐arm splint including (1) X‐lite, a composite of cotton mesh impregnated with a thermoplastic resin (Classic Dispenser White, 125 mm × 10 m, ref: 642540000, Camp Scandinavia AB/Allard Int.); (2) Woodcast, a composite of woodchips and a biodegradable thermoplastic polymer mixture (2 mm Rigid Vented, 2 mm × 145 mm × 800 mm, ref: C1480, Onbone Oy) [26]; (3) PoP, made of calcium sulphate hemihydrate and 17‐thread count cotton gauze fabric (Cellona, 120 mm × 20 m, ref: 25501, Lohmann & Rauscher GmbH); and (4) A 3D‐printed splint made of polypropylene (The 3D‐Print Center at Aarhus University Hospital, in collaboration with Spentys SA/NV), which is a semi‐flexible material with a density of 0.89 g/cm^3^.

**Table 1:** Splint characteristics [3, 6, 7, 9].

Splint	Price^a^	Time to load‐bearing	Heating time to reach formability	Structural weakening due to water exposure^b^
Plaster of Paris (eight layers)^c^ [3, 7]	0.5–2 €	36–48 h Partially after 30 min	<1 min	Yes
Woodcast (2 mm, rigid vented)^d^ [6]	7–10 €	5–10 min Partially after 3–5 min	1.7 min	None/negligible
X‐lite (Classic, two layers)^e^	5–7 €	30 min Partially after 5 min	3 min	Can be cleaned with water
3D‐printed (polypropylene) [9]	57 €	–	–	No

^a^ Price estimate for a 26‐cm‐long splint at the local institution.

^b^ Based on manufacturer information and limited literature.

^c^ Manufacturer User manual, Plaster of Paris (Cellona, ref: 25501, Lohmann & Rauscher GmbH) https://www.lohmann-rauscher.com/dk-da/products/plaster-room/plaster-synthetic-casts/cellona-plaster-of-paris-bandage/ [Accessed 19th October 2023].

^d^ Manufacturer information, Woodcast (Onbone Oy), Woodcast Academy. https://academy.woodcast.com/woodcast-heating-times/, https://academy.woodcast.com/what-is-woodcast/ [Accessed 19th October 2023].

^e^ Manufacturer information, X‐lite (Classic, Camp Scandinavia AB/Allard Int.) https://www.camp.dk/produkter/plast-og-materiale/xlite/xlite-r-classic-ark-og-dispenser-p28469 [Accessed 19th October 2023].

### Splint preparation

#### X‐lite, Woodcast and PoP

**Figure 1:**
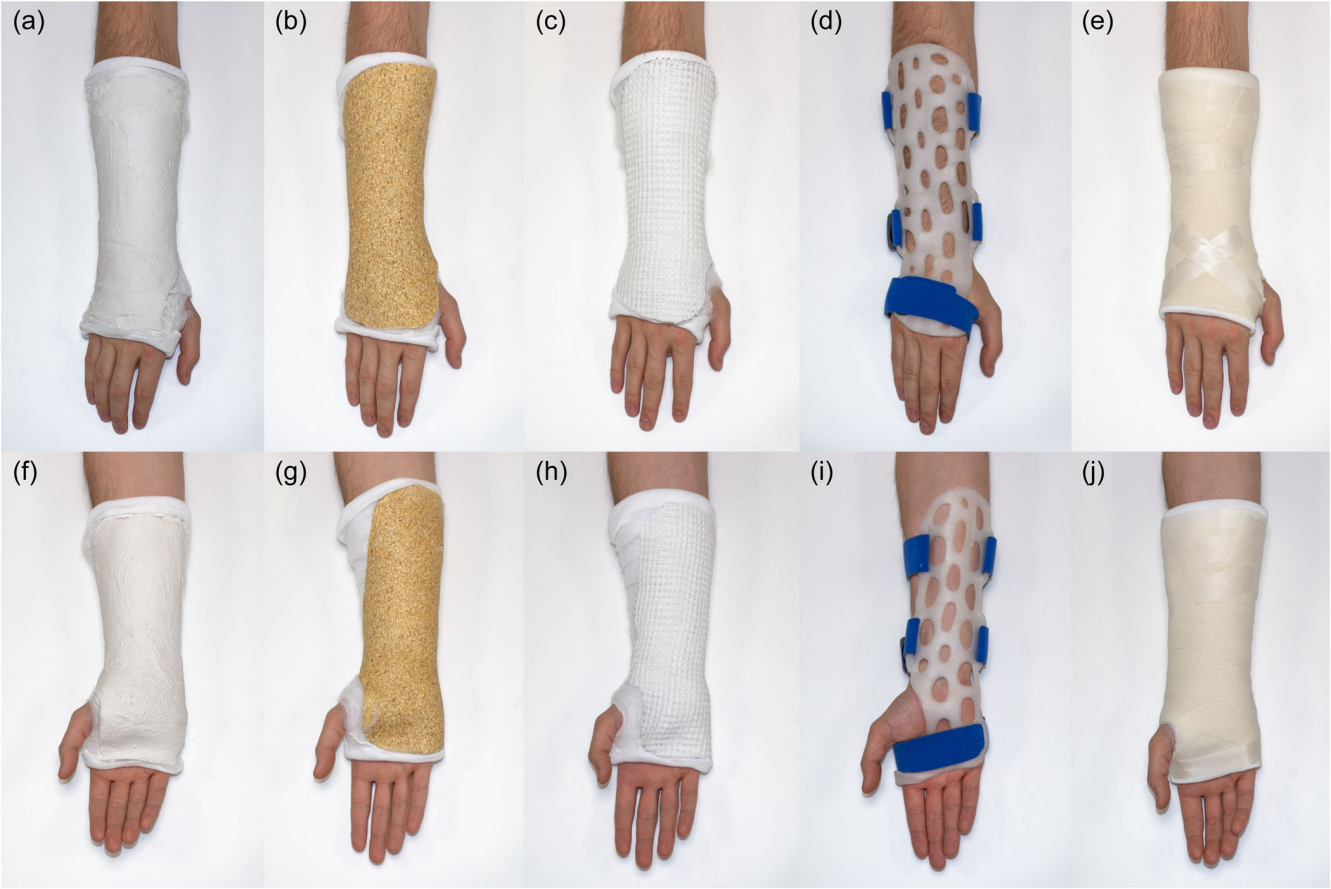
Illustration of splint appearance after application of the volar and dorsal splints. (a–e) Dorsal and (f–j) volar splints: (a, f) Plaster of Paris, (b, g) Woodcast, (c, h) X‐lite, (d, i) 3D‐printed and (e, j) X‐lite with elastic bandage.

To ensure consistency, an experienced orthopaedic nurse (MBA) fabricated all splints and prepared all arm specimens with elastic bandages, wadding and splints (Figure 1). Great care was taken to ensure uniformity in the tightness of the elastic bandage/wadding as well as splint placement and moulding. First, a stockinette was applied to the arm specimens with a hole for the thumb, followed by wadding applied circumferentially with a 50% overlap onto the previous layer, starting 5–15 mm proximal to the knuckles and ending 60–80 mm distal to the elbow. Then, a splint composed of any of the three materials was made and mounted on the arm specimen, moulding it to the contour of either the dorsal side (dorsal splints) or the volar side (volar splints) of the forearm. A roll of elastic bandage was used to maintain a natural wrist position during moulding and rest period. Finally, an elastic bandage was applied with a 50% overlap and secured with tape.

The splints were created according to the respective manufacturer's recommendations. For X‐lite, the material was immersed in 70°C water for 3 min, folded along the width axis to create a two‐layer splint, moulded to the arm specimen and cut to the appropriate size (Table 2). The X‐lite splints were left to harden for a minimum of 30 min before testing. For Woodcast, the material was heated (Onbone Express Heater, ref: A002, Onbone Oy) at 65°C for 100 s. Then, the splints were moulded to the arm specimens and customized to specimen size. The Woodcast splints were left to rest for a minimum of 30 min before testing. PoP was folded along the width axis to create an 8‐layer splint. After a brief immersion in 21°C water, followed by gentle shaking to remove excessive water, the material was manually laid flat and smoothed. After the splint was moulded to the arm specimen and cut to size, it was left to rest for 24 h before testing.

**Table 2:** Mean (range) splint length and width (not including wadding/gaze).

Material	Dorsal splints		Volar splints	
	Length (mm)	Width (mm)	Length (mm)	Width (mm)
Plaster of Paris (*n* = 10)	256 (249–263)	127 (120–132)	263 (252–275)	127 (123–129)
Woodcast (*n* = 10)	258 (243–269)	154 (150–162)	257 (249–264)	153 (144–162)
X‐lite (*n* = 10)	285 (274–300)	133 (128–136)	277 (266–300)	139 (136–143)
3D‐printed (*n* = 10)	265 (230–296)	109 (93–126)	269 (242–297)	105 (87–135)

#### 3D‐printed splints

The 3D‐printed splints were created using individual 3D models of each arm specimen. First, the arm specimen was scanned using a portable 3D scanner (Structure Sensor Mark II, XRPro LLC, USA) using infrared pattern emission technology. The 3D data were processed (Spentys, V1.0.11, Belgium) to create 3D models of the individual arm specimens. Then, these models were used to create personalized 3D‐printed volar and dorsal splints. Openings in the splinting material were manually customized to each splint to allow ventilation and facilitate weight reduction of the splints. 3D printing was performed by Spentys SA/NV (Belgium). Key print settings: Material: polypropylene, layer height: 0.2 mm, infill pattern: Gyroid, printing temperature: 215°C, print speed: 25 mm/s, support structure: Tree/organic. Postprocessing of the splints was performed with a scalpel, pliers and sandpaper. Finally, the volar and dorsal 3D‐printed splints were equipped with adjustable elastic Velcro straps and secured to the arm specimens, as recommended for clinical use.

### Test setup and fixture device

**Figure 2:**
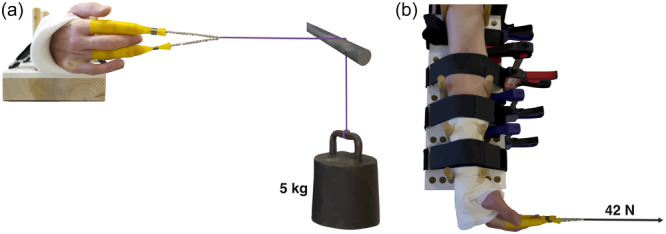
Illustration of the experimental test setup. The radiolucent fixture and an arm exposed to 42 N load in wrist flexion viewed from (a) the front and (b) above.

All arm specimens were sequentially equipped with each of the four different splints and tested in a custom‐made radiolucent fixture designed to allow passive loaded wrist flexion and extension in a standardized way (Figure 2). Sequentially, static radiographic images of all arm specimens with each splint type were recorded in the following order: Neutral, flexion and extension.

The splints were dismounted from the arm specimens before new splints were fabricated and mounted and radiographic images were recorded. Testing was conducted over three days in the following sequence: Day 1: Volar Woodcast, volar 3D‐printed and dorsal Woodcast. Day 2: Dorsal PoP, volar X‐lite, dorsal X‐lite and dorsal 3D‐printed. Day 3: Volar PoP.

Before testing, the arm specimens were rigidly fixed in the fixture in a neutral forearm position (thumb and humerus oriented in the same direction, pointing up), defined as a complete overlay of the ulna and radius on lateral radiographs. The distal fixation point was approximately 2 cm proximal to Lister's tubercle, which allowed for passive wrist flexion and extension as permitted by the splint. Fixation was performed with adjustable wooden rods, while foam wedges secured the ventral and dorsal aspect of the antebrachium rigidly. In addition, the forearm was secured to the fixture with Velcro straps, and the brachium was fastened with a strap. Prior to load application, the correct positioning of the arm specimens was evaluated via radiography.

Two finger traps, attached to the second and fourth finger, were connected to a 5‐kg weight suspended over a metal rod using a cotton string, exerting a force of 42 N (Figure 2). The load was chosen since previous cadaveric studies have used bending forces of 4.5–80 N in distal radius fracture models [22, 23, 25, 27, 28]. The wrist was oriented orthogonally to the forearm in the sagittal plane of the arm specimens during both wrist flexion and extension testing. Static radiographic images were made in lateral projection using a digital Adora DRFi system (NRT X‐Ray A/S) with the following exposure parameters: X‐ray voltage of 65 kV and current of 5 mAs and a source image receptor distance (SID) of 1.1 m.

### Radiographic evaluation

**Figure 3:**
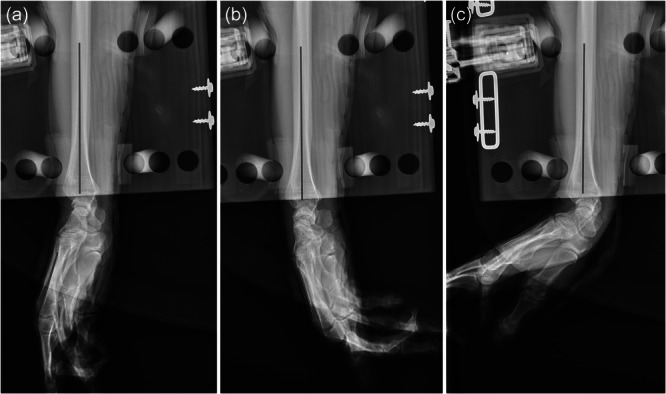
Radiographic measurements of an arm specimen. Arm specimen with a volar 3D‐printed splint in (a) neutral, (b) flexion and (c) extension.

Evaluation of all radiographic images was performed by two independent observers (HCR and JGL). Blinding for the splinting material was not possible since the splinting materials were visible on the radiographs. To establish consensus, a specialist in orthopaedic hand surgery (MS) provided instruction on how to perform the measurements to the two observers using 20 randomly selected images prior to the actual evaluations. The length axis of the radius and third metacarpal was marked on all radiographs, and the angle between these lines' length axis was given by the utilized clinical software (Enterprise Imaging v.8.1.2 SP7.10, Agfa‐Gevaert NV) (Figure 3). The mean wrist flexion/extension was defined as the difference in measured angles on wrist flexion/extension recordings relative to the neutral recording. The angles were measured by both observers on all radiographic images. In total, 240 radiographic images were recorded and evaluated.

The first line, representing the relative position (length axis) of the radius, was marked from the centre of the diaphysis as the proximal point and extending distally to the centre of the articulate surface of the radiocarpal joint. The second line, representing the relative position of the third metacarpal (length axis), was marked along the dorsal margin of the diaphysis of the third metacarpal, starting distal to the metacarpal basis and extending proximal to the metacarpal head. Attention was given to ensuring the lines were parallel with the bone margins, especially when the third metacarpal bone's margins were ambiguous. The third metacarpal bone was chosen since it is the central bone of the hand, and traction was applied to the second and fourth fingers.

### Sheet preparation for mechanical testing

**Figure 4:**
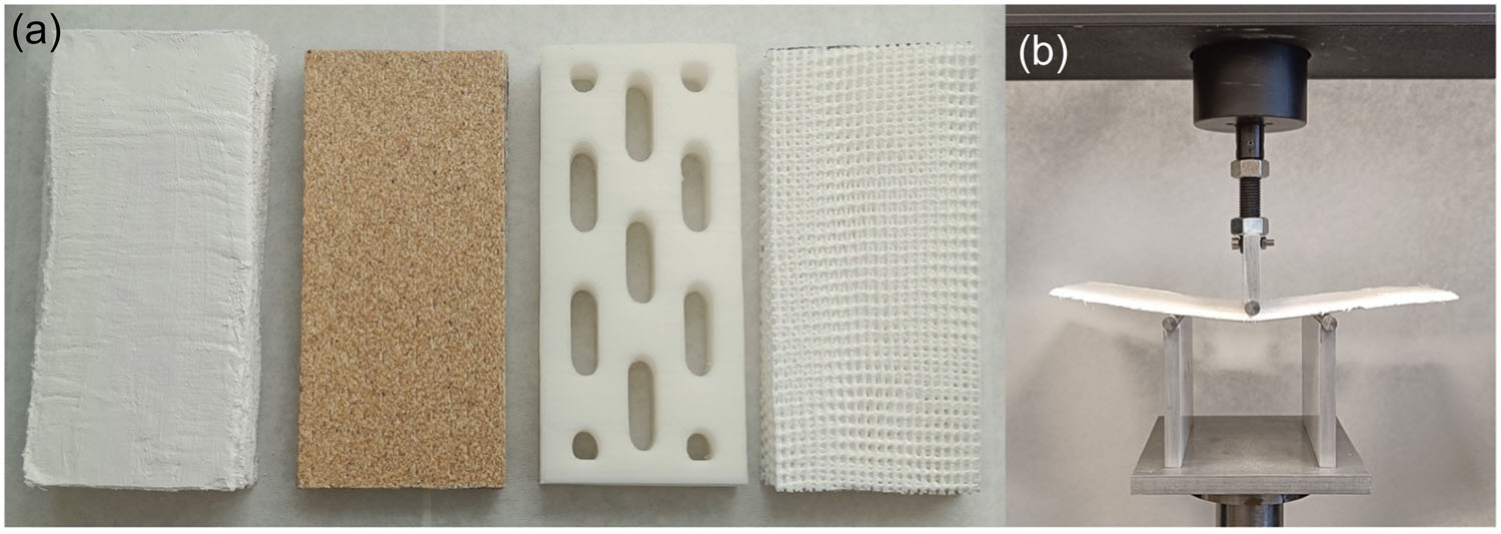
Splint sheets and mechanical test setup. (a) Sheets of the splinting material from left: Plaster of Paris, Woodcast, 3D‐printed and X‐lite. (b) Setup of the three‐point bending test.

Ten standardized rectangular sheets of each splinting material were created utilizing the same technique as described above, but the sheet specimens were moulded flat on a table and left for hardening for at least 1 week (Table 3 and Figure 4). The 3D sheets were designed to match the 3D splints using 3‐Matic (Materialise NV, Belgium) and 3D‐printed using the method described above.

**Table 3:** Sheet dimensions and weight.

Material	Length (mm)	Width (mm)	Height (mm)	Weight (g)
Plaster of Paris	261 (0.7)	119 (1.4)	5 (0.3)	157 (3.1)
Woodcast	260 (0.8)	120 (1.0)	2 (0.1)	70 (2.2)
X‐lite	260 (1.8)	120 (0.7)	3 (0.2)	54 (1.9)
3D‐printed	259 (0.7)	119 (0.5)	4 (0.1)	65 (0.5)

*Note*: Mean (SD).

Abbreviation: SD, standard deviation.

### Evaluation of mechanical properties of the splinting material

A three‐point bending test was performed using a materials testing machine (Instron model 5566) equipped with a 10‐kN load cell. The sheets were placed horizontally and centred on a custom‐made testing jig resting on two cylindrical, rounded bars (Ø 10 mm) separated by 100 mm (Figure 3). Vertical load was applied at the midpoint of each sheet with a third rounded bar (Ø 10 mm) at a constant deflection rate of 10 mm/min until the material fractured. During the testing, data were recorded using the software provided with the materials testing machine (Merlin, version 3.21, Instron Ltd), producing individual load‐deflection data. The mechanical properties evaluated were (1) extrinsic flexural rigidity (stiffness, N/mm), (2) force to fracture (*F*_max_, N) as a measure of extrinsic flexural strength and (3) deflection at maximum extrinsic fracture load (Δx at *F*_max_, mm), that is, how much the material bent before it failed. The force to fracture was defined as the first local maximum of the load‐deformation curve where the material properties were compromised. The mechanical data were analysed using in‐house developed software (by JST).

### Statistical analysis

No pre‐study power calculation was performed. However, comparable studies, assessing the capability of different casting materials to stabilize distal radius fractures, had sufficient power to detect differences with <10 specimens [15, 25].

Statistical analysis of the wrist flexion/extension data was performed using STATA (version 17.0, StataCorp), and a mixed‐effects model with crossed random effects was used to analyse the data. The random effects associated with the specimen and the crossed random effects between specimen and splint type, as well as specimen and flexion/extension were included in the model. Restricted maximum likelihood was employed to ensure unbiased variance component estimates, and the Kenward Rogers approximation was used to estimate degrees of freedom due to the low sample size. Pairwise comparisons of estimates were performed with small sample statistics, significance level was defined as *p* ≤ 0.05.

The mechanical material data were analysed using Prism (version 9.5.1, GraphPad Software LLC). A one‐way ANOVA followed by a Fisher's least significant difference post hoc test was performed on the *F*_max_ data. Material stiffness and deflection at *F*_max_ were analysed using a Welch's ANOVA test followed by unpaired *t* tests with Welch correction due to conditional heteroscedasticity.

Inter‐observer agreement and reliability were assessed using all recorded radiographs. To determine intra‐observer agreement and reliability, 30 randomly picked images, evenly distributed between splint type and specimens, were reanalysed a week after the original evaluation. The inter‐observer and intra‐observer agreement and reliability were evaluated using mean difference with standard deviation (SD), 95% limits of agreement (95% LoA) and intraclass correlation coefficient (ICC) with 95% CI. ICC was determined based on a two‐way mixed effects model, mean rating and both absolute agreement and consistency of agreement.

## RESULTS

### Radiographic wrist flexion/extension data

All specimens and radiographic images were used for analysis, except for one radiological image of a specimen in extension that was lost in data management.

#### Dorsal splints

**Figure 5:**
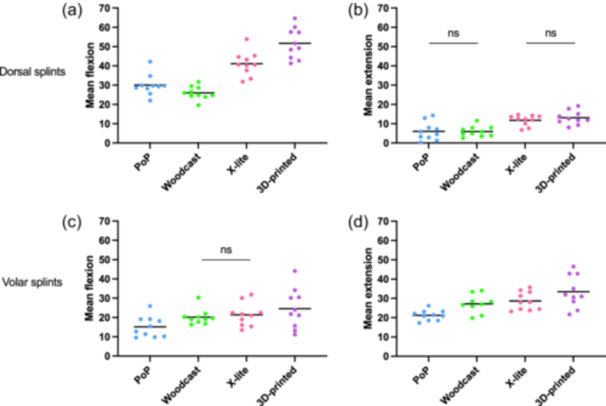
Mean flexion and extension for all splints. (a) Dorsal splint wrist flexion, (b) extension, (c) volar splint flexion and (d) extension for all splinting materials. Only statistical non‐significance (*p* > 0.05) is shown (ns), all other comparisons are significant. PoP, plaster of Paris. Mean values and individual data points.

The 3D‐printed splint allowed the highest mean wrist flexion at 53° (95% CI: 49°–57°) followed by X‐lite at 42° (95% CI: 39°–46°). The lowest mean wrist flexion was observed for Woodcast at 27° (95% CI: 23°–30°), while PoP presented with mean wrist flexion of 31° (95% CI: 28°–35°) (Table 4 and Figure 5). A similar pattern was found for the mean wrist extension with dorsal splints. The highest mean extension was seen for the 3D‐printed splint with 13° (95% CI: 10°–17°), followed by X‐lite with a mean of 12° (95% CI: 8°–16°), while the lowest mean wrist extension was found for Woodcast and PoP, which both displayed a mean wrist extension of 6° (95% CI: 3°–10°).

**Table 4:** Mean wrist flexion and extension (95% CI) for all splints.

Material	Dorsal splints		Volar splints	
	Flexion (°)	Extension (°)	Flexion (°)	Extension (°)
Plaster of Paris	30 (27–34)	6 (3–10)	15 (12–19)	21 (18–25)
Woodcast	26 (22–30)	6 (2–10)	20 (17–24)	28 (24–31)
X‐lite	41 (38–45)	12 (8–15)	21 (18–25)	29 (25–32)
3D‐printed	52 (48–55)	13 (10–17)	25 (21–28)	33 (30–37)

Abbreviation: CI, confidence interval.

#### Volar splints

The 3D‐printed splint allowed for the highest mean wrist flexion at 25° (95% CI: 22°–29°), followed by X‐lite at 21° (95% CI: 18°–25°), Woodcast at 20° (95% CI: 17°–24°) and PoP at 16° (95% CI: 13°–20°). A similar pattern was observed for mean wrist extension, where the 3D‐printed splint displayed the highest mean extension at 34° (95% CI: 30°–38°), while X‐lite and Woodcast both showed 29° (95% CI: 26°–33°) and PoP exhibited the lowest mean wrist extension at 22° (95% CI: 19°–26°).

#### Observer agreement and reliability

For both investigators, the intra‐observer mean difference in wrist flexion/extension was −1.0° (95% LoA: −4° to 3°) or below (Table 5). The inter‐observer mean difference for wrist flexion was −0.2° (95% LoA: −5° to 5°) and for wrist extension it was 0.4° (95% LoA: −5° to 6°). The intra‐observer absolute agreement ICC was ≥0.97 (95% CI: 0.89–0.99) for both observers and the inter‐observer ICC was ≥0.98 (95% CI: 0.98–0.99) for both flexion and extension (Table 5). The consistency of agreement ICC was ≥0.97 (95% CI: 0.88–0.99) for intra‐observer and ≥0.98 (95% CI: 0.98‐0.99) for inter‐observer reliability for both flexion and extension.

**Table 5:** Agreement and reliability for wrist flexion and extension.

Agreement and reliability	Wrist flexion				Wrist extension			
	ICC (95% CI)		MD (SD) (°)	95% LoA	ICC (95% CI)		MD (SD) (°)	95% LoA
Intra‐observer 1	1.00 (0.99–1.00)		−1.0 (1.4)	(−2 to 2)	1.00 (0.99–1.00)		−0.2 (1.0)	(−3 to 4)
Intra‐observer 2	1.00 (0.99–1.00)		−1.0 (1.5)	(−4 to 3)	0.97 (0.89–0.99)		−0.7 (3.7)	(−6 to 6)
Inter‐observer 1/2	0.99 (0.99–0.99)		−0.2 (2.4)	(−5 to 5)	0.98 (0.98–0.99)		−0.4 (2.8)	(−5 to 6)

*Note*: Intraclass correlation coefficient (ICC) (two‐way mixed effects, absolute agreement, mean‐rating), mean difference (MD) and 95% limits of agreement (95% LoA) for wrist flexion and extension.

### Mechanical properties of the splinting material

**Figure 6:**
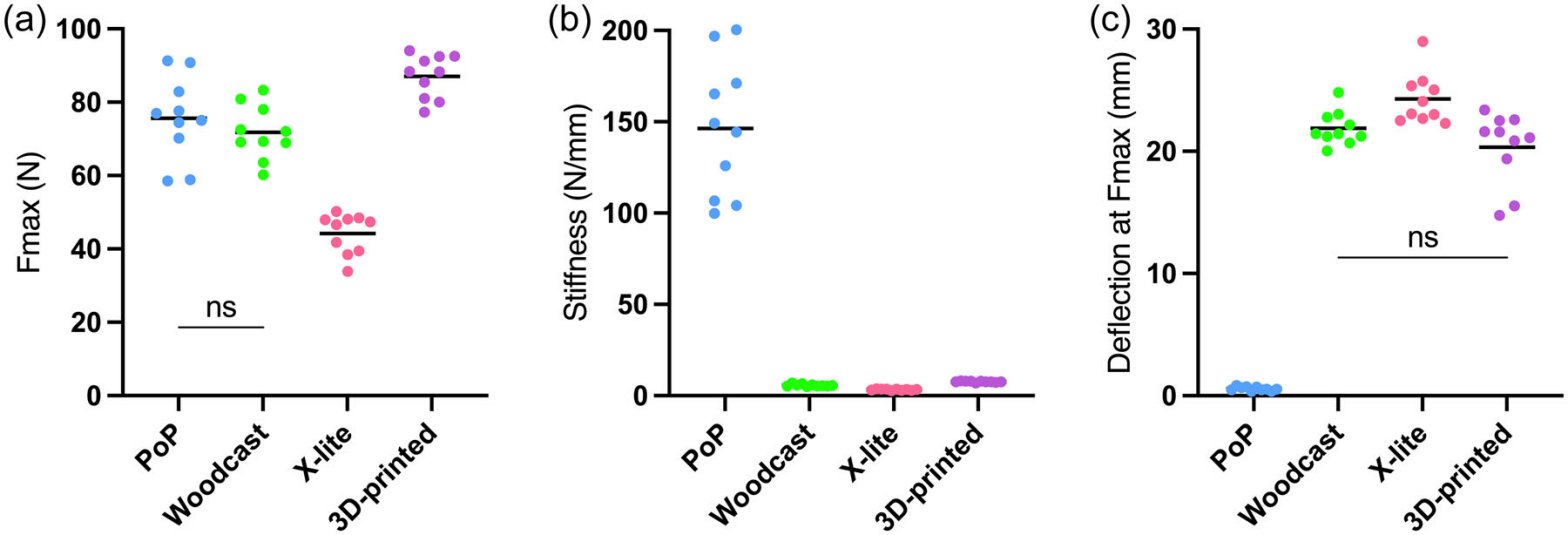
Material properties. (a) Force to fracture (*F*_max_), (b) stiffness and (c) deflection at *F*_max_ determined by a three‐point test for all splinting materials. Only statistical non‐significance (*p* > 0.05) is shown (ns), all other comparisons are significant. PoP, plaster of Paris. Mean values and individual data points.

Of the tested splinting materials, the 3D‐printed (polypropylene) sheets had the highest mean *F*_max_, while X‐lite demonstrated the lowest *F*_max_ (Table 6 and Figure 6). There was no difference in mean *F*_max_ between the PoP and Woodcast. Notably, PoP displayed a mean stiffness of 146 (95%CI: 120–173) N/mm, which was substantially higher than that of the other splinting materials, which showed mean stiffnesses of 3.3 (95% CI: 3.1–3.6) N/mm (X‐lite), 5.7 N/mm (95% CI: 5.2–6.1) (Woodcast) and 7.7 (95% CI: 7.4–7.9) N/mm (3D‐printed). The opposite was seen for mean deflection at *F*_max_, as X‐lite displayed the highest mean deflection at failure, followed by Woodcast and the 3D‐printed material, while PoP failed at the lowest deflection.

**Table 6:** Mean (95% CI) material stiffness, *F*_max_ and deflection at *F*_max_ of the different splinting materials.

Material	Stiffness (N/mm)	*F*_max_ (N)	Δ *x* at *F*_max_ (mm)
Plaster of Paris	146 (120–173)	76 (68–84)	0.6 (0.5–0.7)
Woodcast	5.7 (5.2–6.1)	72 (67–77)	22 (21–23)
X‐lite	3.3 (3.1–3.6)	44 (40–48)	24 (23–26)
3D‐printed	7.7 (7.4–7.9)	87 (83–91)	20 (18–22)

Abbreviations: CI, confidence interval; F_max_, force to fracture.

## DISCUSSION

In the present study, a standardized cadaveric setup was used to evaluate the wrist‐stabilizing properties of four different splinting materials: X‐lite (classic, two layers), Woodcast (2 mm rigid vented), PoP (eight layers) and custom‐made 3D‐printed (polypropylene). For the volar splints, PoP had the highest capability to resist wrist flexion and extension. For the dorsal splints, Woodcast exhibited a lower wrist flexion, and a similar wrist extension compared to PoP. The 3D‐printed splints—both volar and dorsal—exhibited the highest mean wrist flexion and extension. The relative difference in the mean wrist flexion/extension range for the volar and dorsal splints varied from 7° to 26°. The clinical significance of these differences is difficult to determine, given that the degree and technique of immobilization required for optimal healing of distal radius fractures in conservative treatment remain unclear [13, 29]. This extends to both the optimal choice of splinting material and the general design of the cast/splint.

One study evaluated circumferential casting, volar‐dorsal splinting and a modified sugar‐tong splinting in displaced distal radius fractures requiring closed reduction [11]. The study found no differences in loss of reduction at 3–4 weeks or in functional outcomes at 8 weeks and 6 months follow‐up. The authors noted that these results could be attributed more to the fracture pattern and the quality of the initial reduction than to the actual immobilization technique. These findings challenge the notion that greater wrist immobilization leads to better radiological outcomes and a reduced loss of reduction. However, the current literature sparsely evaluates below‐elbow splints and is limited by methodological issues, including small sample sizes and heterogeneity in the reported interventions and outcomes [13, 17]. Despite these limitations, adequate fracture stabilization, though poorly defined, is still considered crucial for bone healing [29], making the clinician's empirical experience essential in selecting the appropriate immobilization technique [11, 17, 19].

Several mechanical properties should be considered when defining the best immobilization material. The flexural material strength may not be the most important parameter, as splint breakage is seen in ≤6% of splints of various materials, including Woodcast and PoP [5, 12, 20]. However, a splinting material with high stiffness may—theoretically—stabilize a bone fracture to a larger extent than a material with lower stiffness. In the three‐point bending test, a distinctly higher material stiffness was observed for PoP compared to the remaining splinting materials. Interestingly, the material stiffness of the remaining splinting materials was comparable, highlighting the complexity of comparing standardized sheets of splinting material to the wrist‐stabilizing properties of three‐dimensional real‐life splints.

When choosing an optimal splinting material, it is important to consider features other than mechanical material properties such as price, time ease of application, weight and comfort, as a material with high stiffness likely provides less user comfort than a more flexible material. In particular, PoP has the disadvantage of being susceptible to strength loss when exposed to water; however, it is substantially cheaper and stiffer than the other splinting materials [3]. On the other hand, in situations where stiffness is less indicated (e.g. painful tenosynovitis), comfort may be more important to consider. The tested 3D‐printed splint holds the potential to improve patient comfort, but the wrist‐stabilizing properties were inferior to the other splints tested and the manufacturing time and price were markedly higher.

### Observer agreement and reliability

The mean difference between inter‐ and intra‐observer measurements of the wrist flexion/extension was within the range of −1.0° to 0.4°. This was considered acceptable as the mean difference and 95% LoA estimates were lower compared to a previous study investigating dorsal/volar tilt observer agreement in a cadaveric setup [10]. The absolute intra‐observer and inter‐observer reliability of ≥0.97 were deemed satisfactory since an ICC of >0.9 has been proposed to indicate excellent reliability [18]. The consistency of agreement ICC is, by definition, equal to or higher than absolute agreement ICC and the relationship between the two can be used as a marker of systematic bias [2, 21]. In the present study, the consistency of agreement ICC was similar to the absolute agreement ICC for both intra‐observer and inter‐observer agreement indicating a low degree of bias.

### Limitations

Wrist stability was assessed using cadaveric arms with no fractures, and the external validity is therefore limited to immobilization of wrist motion without any influence of muscle contraction. Due to radiation exposure and ethical reasons, the experiment could not have been performed in patients (particularly distal radius fracture patients). We considered a distal radius fracture model but struggled to find a way to ensure the equivalent fracture position at baseline between specimens and for repeated testing of the specimens.

The forces exerted on the wrist during active wrist movement are not well understood, but axial compressions of 100 N during light wrist movement and not over 250 N, including the movement of the digits, have been suggested [1, 27]. The load applied in the test setup was external and static, making it less representative of active wrist movement in a patient, but clinically comparable to the force experienced when lifting or pushing with a 42 N load. Moreover, it should be noted that the load exerted on the splints was affected by the fixture device, bandages and the arm specimens themselves. Notably, the elastic bandages, which were not applied to the 3D‐printed splints to mirror clinical practice, and their application affected wrist movement and could potentially introduce a bias. To address this, a crossed design and standardized setup were used for splint comparison, providing a proxy for the mechanical capability of the splinting materials to stabilize the wrist when applied as either volar or dorsal splints.

While differences in the application of the splints to the arm specimens may have caused variation or unwanted bias, these effects were minimized by the fact that a single registered orthopaedic nurse applied all splints. The low ICCs observed indicate a low level of bias for the radiological measurements. Moreover, the mechanical three‐point bending test of the splinting material was substantially less biased by application but showed similar variations in the outcome compared to the radiographic wrist angle test.

The arm specimens were stored at −18°C for 1–3 years before testing, and each arm specimen was tested multiple times over three consecutive days. This may have affected the tissue and tendon biomechanics and introduced potential sources of bias [4].

## CONCLUSION

In summary, the dorsal splints were better at resisting wrist extension, while the volar splints were better at resisting wrist flexion. PoP (eight layers) showed superior wrist‐stabilizing properties both applied as a volar and dorsal short arm splint, followed sequentially by Woodcast (2 mm, rigid vented), X‐lite (classic, two layers) and 3D‐printed (polypropylene). The mechanical properties of the Woodcast, X‐lite and 3D‐printed sheets were similar, while PoP exhibited distinctly higher material stiffness and lower deflection at *F*_max_. These findings can be used to support evidence‐based condition‐specific recommendations for situations where immobilization of the wrist is indicated. If greater wrist immobilization is required for treating wrist disorders, such as fractures, PoP may be preferable. However, factors including comfort, ease of application and time to load‐bearing may favour the other splinting materials.

## AUTHOR CONTRIBUTIONS

*Conceptualization*: Maiken Stilling, Maya Bang, Jesper Skovhus Thomsen, Hans Christian Rasmussen and Lars Lindgren. Methodology: Maiken Stilling, Maya Bang, Jesper Skovhus Thomsen, Mads Kristian Duborg Mikkelsen, Hans Christian Rasmusse, Lars Lindgren and Mats Bue. *Formal analysis and investigation*: Hans Christian Rasmussen, Jesper Skovhus Thomsen, Johanne Gade Lilleøre, Josephine Olsen Kipp and Maiken Stilling. *Writing—original draft preparation*: Hans Christian Rasmussen. *Writing—review and editing*: Hans Christian Rasmussen, Maya Bang, Johanne Gade Lilleøre, Josephine Olsen Kipp, Lars Lindgren, Annemarie Brüel, Mats Bue, Mads Kristian Duborg Mikkelsen, Jesper Skovhus Thomsen and Maiken Stilling. *Supervision*: Josephine Olsen Kipp, Mats Bue, Jesper Skovhus Thomsen, Maya Bang and Maiken Stilling. All authors read and approved the final manuscript.

## CONFLICT OF INTEREST STATEMENT

The authors declare no conflict of interest.

## ETHICS STATEMENT

The study was performed in accordance with the Declaration of Helsinki and its later amendments or comparable ethical standards. The study was carried out in agreement with national and international law and with approval by the Central Denmark Region Committees on Health Research Ethics (license No. 1‐10‐72‐144‐23). The authors affirm that informed consent was achieved for publication of the images used in Figures 1 and 2.

## Data Availability

The data sets used and/or analyzed during the current study are available from the corresponding author upon reasonable request.
